# SUMOylation regulates the localization and activity of Polo-like kinase 1 during cell cycle in the silkworm, *Bombyx mori*

**DOI:** 10.1038/s41598-017-15884-7

**Published:** 2017-11-14

**Authors:** Zhiqing Li, Qixin Cui, Jian Xu, Daojun Cheng, Xiaoyan Wang, Bingqian Li, Jae Man Lee, Qingyou Xia, Takahiro Kusakabe, Ping Zhao

**Affiliations:** 1 State Key Laboratory of Silkworm Genome Biology, Southwest University, Chongqing, China; 2 Chongqing Engineering and Technology Research Center for Novel Silk Materials, Chongqing, China; 3 Laboratory of Insect Genome Science, Kyushu University Graduate School of Bioresource and Bioenvironmental Sciences, Fukuoka, Japan

## Abstract

Polo-like kinase 1 (Plk1) is a crucial cell cycle regulator by its specific localization and activity during cell cycle. It has been shown that the phosphorylation and ubiquitylation of Plk1 are required for its own activation and localization. Here, we report that SUMOylation regulates the activity of Plk1 in the lepidopteran insect of *Bombyx mori*. In the absence of SUMOylation, it causes the lost localization of Plk1 on centrosomes and kinetochores, as well as an uneven distribution in midzone. We further identify that the putative SUMOylation site of *Bombyx* Plk1 at lysine 466 is required for its localization on centrosomes, and K466 mutation in Plk1 could influence its interaction with Smt3/Ubc9 complex. These findings are also confirmed by *Drosophila* Polo and human Plk1, which together reveals a conserved role of Plk1 SUMOylation in mammals. Moreover, conjugation of Smt3 to Plk1 SUMOylation mutant promotes its localization on centrosomes and kinetochores, and rescues functional defects of chromosome alignment in cells depleted of endogenous Plk1. Altogether, the present data indicate that the SUMOylation of Plk1 could participate in proper chromosome alignment and segregation during mitosis, and provides a novel layer for the regulation of Plk1 localization and activity throughout cell cycle.

## Introduction

Polo-like kinase 1 (Plk1), as a mitotic kinase, is one of the most important regulators for cell cycle progression in various eukaryotic organisms from yeast to mammals^1–3^. It has been considered that the regulation for cell cycle by Plk1 activity is largely ascribed to the proper localizations of Plk1 to the accurate fraction of subcellular structure so as to exert its function^3–5^. Consistent with this, Plk1 is dynamically located in various structures when cells progress through the cell cycle^4–6^. For example, during interphase, Plk1 is specifically localized to the centrosomes and is involved in the regulation of centrosome maturation and separation as well as bipolar spindle formation. Recruitment of Plk1 to the kinetochores in the early mitosis will facilitate the attachment of kinetochores with microtubules, which is implicated in modulating chromosome alignment and segregation. Further, the localization of Plk1 to the midzone and midbody late in mitosis has shown the other roles of Plk1 in cytokinesis^3–6^.

Consistent with the functions of Plk1 in the cell cycle, expression of Plk1 has been reported to be elevated in human cancers, which contributes to the formation and progression of tumors^7–9^. It is thus thought as a negative prognostic marker and emphasized the importance of Plk1 in tumorigenesis. Indeed, silencing of Plk1 levels by RNAi or inhibition of Plk1 activity by specific inhibitor is capable of decreasing the growth of most cancer cells, influencing mitotic spindle formation, and promoting apoptosis^9–12^. As such, the control of Plk1 activity has been an attractive candidate for anticancer drugs in the administration of human tumors^9,10,13^.

There are several ways for the regulation of Plk1 functions, including the control of its expression, the change of localization, and the regulation of activity^2^. The expression of Plk1 is regulated in a cell cycle-dependent profile with an increase in G2 phase and a high peak in the early stage of mitosis. Plk1 could thus be recruited to centrosomes in interphase, and localized on kinetochores, centromeres, spindle midzone, and midbody during mitosis. It has been recently reported that the ubiquitylation-dependent localization of Plk1 in mitosis modulates its recruitment to kinetochores and regulates the faithful alignment and segregation of chromosomes^14^. Another layer for Plk1 regulation is its activation. In G2 phase, the aurora kinase family member of Aurora-A is localized on centrosomes and able to phosphorylate Plk1 at a conserved threonine residue T210, which induces the activation of Plk1^2,15,16^. When cells enter mitosis, the kinase of Aurora-B is also responsible for T210 phosphorylation and Plk1 activation^2,17,18^.

Similar to the ubiquitylation, SUMOylation occurs at the same lysine residue of substrate proteins, which are involved in diverse biological pathways including protein localization, gene transcription, cell cycle progression, and genomic stability^19–22^. In this study, we identified BmPlk1 as a substrate of the SUMOylation system in the silkworm *Bombyx mori*, which could interact with BmSmt3/BmUbc9 complex to regulate the localizations of BmPlk1 on centrosomes and kinetochores. The further mutation analysis of BmPlk1 at several putative SUMOylation sites revealed the probable lysine 466 is able to participate in the SUMOylation modification. Importantly, the site of BmPlk1 lysine 466 is much conserved with the human Plk1 lysine 492 that has been confirmed as an ubiquitylation site. All these data thus provides a very interesting crosstalk between ubiquitylation and SUMOylation on the same protein target, and gives a novel layer for the regulation of Plk1 localization and activity mediated by the SUMOylation signaling during the cell cycle.

## Results

### Identification of a silkworm protein kinase that is functionally conserved with *Drosophila* Polo and human Plk1

**Figure 1:**
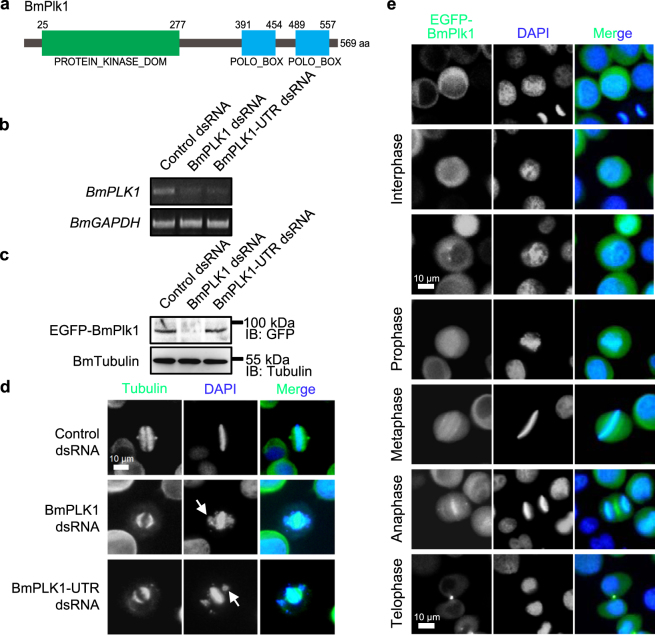
Identification of silkworm Plk1 that is functionally conserved with *Drosophila* Polo and human Plk1. (**a**) A schematic representation of the domain structure of BmPlk1. (**b**) Semi-quantitative PCR assay of RNAi efficiency for *BmPLK1* in cultured silkworm cells treated by control dsRNA, BmPLK1 dsRNA, or BmPLK1-UTR dsRNA. The expression of *BmGAPDH* gene was used as a loading control. Cropped gels are displayed and full-length gels can be found in Supplementary Fig. S5a,b. (**c**) In the EGFP-BmPlk1 expressing cells, BmPLK1 dsRNA could decrease both the endogenous and exogenous expression of *BmPLK1*, while BmPLK1-UTR dsRNA only affected the endogenous *BmPLK1*. The level of BmTubulin was used as a loading control. IB represents the immunoblotting. Cropped blots are displayed and full-length blots can be found in Supplementary Fig. S5c,d. (**d**) Cells were treated with the indicated dsRNAs and analyzed by immunofluorescence microscopy. The mitotic spindle was stained with anti-Tubulin antibody (green) and nucleus DNA was visualized by DAPI (blue). Arrows indicated the misaligned and lagging DNA. Scale bar, 10 µm. (**e**) Dynamic localization of EGFP-BmPlk1 during the cell cycle in cultured silkworm cells. Scale bar, 10 µm.

Plk1 performs diverse roles throughout the cell cycle. In order to isolate the ortholog of Plk1 and investigate the function in the model lepidoptera species of silkworm, we obtained a full-length sequence from the silkworm genome based on the amino acid sequence of *Drosophila* Polo and human Plk1, and we designated the gene as silkworm *PLK1* (*BmPLK1*). The nucleotide sequence of *BmPLK1* contains an open reading frame of 1710 nt and encodes a protein of 569 aa. Comparison of the deduced amino acid sequence of silkworm Plk1 with other orthologs from various species revealed a high identity and a similar overall structure including one catalytic domain and two polo-box domains (Fig. 1a, Supplementary Fig. S1), suggesting that the silkworm Plk1 may share an evolutionarily conserved function with *Drosophila* Polo and human Plk1.

To decipher the role of silkworm Plk1, we carried out RNAi experiments in cultured silkworm cells by using two different dsRNAs that target the gene body and 3′ untranslated region (UTR) of *BmPLK1*, respectively. Upon knockdown of *BmPLK1* by these dsRNAs, the levels of *BmPLK1* were clearly decreased (Fig. 1b), and moreover, overexpression of EGFP-fused BmPlk1 could be specifically inhibited by BmPLK1 dsRNA but not by BmPLK1-UTR dsRNA (Fig. 1c), which together showed the efficient RNAi for *BmPLK1* in the cultured silkworm cells. We then examined the effects of *BmPLK1* RNAi on cell growth and cell division. As shown in Fig. 1d and Supplementary Fig. S2, depletion of *BmPLK1* notably inhibited the growth of silkworm cells, induced cell apoptosis, and affected chromosome congression and segregation in mitosis, which is consistent with the phenotype from *Drosophila* Polo and human Plk1 RNAi.

Plk1 has been shown multiple localizations during the cell cycle. To examine the localization of BmPlk1 in silkworm cells, we established a stable cell line that expresses the EGFP-fused BmPlk1. As shown in Fig. 1e, the signals of EGFP-BmPlk1 could be observed at cytoplasm and centrosome during interphase, and localized on centrosomes, kinetochores, centromeres, spindle midzone, and midbody following the progression of the mitosis. This wide distribution of BmPlk1 during the cell cycle showed the diverse roles of BmPlk1 plays in silkworm, indicating conserved functions of Plk1 in regulating centrosome maturation, chromosome alignment, spindle assembly, and cytokinesis among different species.

### SUMOylation may regulate chromosome behaviors via BmPlk1 during mitosis

**Figure 2:**
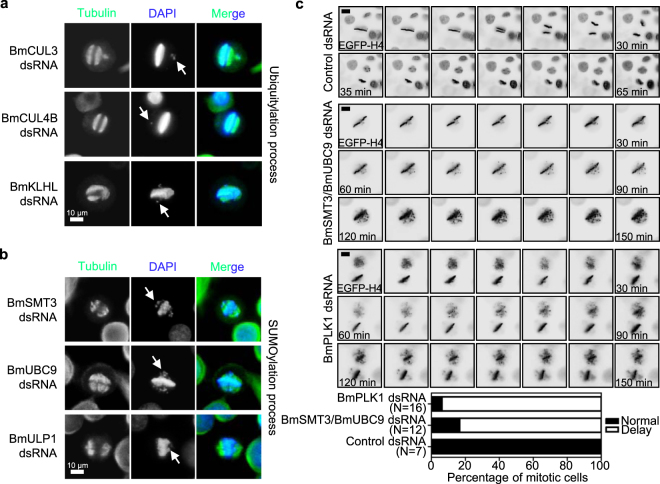
SUMOylation may regulate chromosome behaviors via BmPlk1 during mitosis. (**a** and **b**) RNAi of ubiquitylation genes and SUMOylation genes induced abnormal mitotic spindle in cultured silkworm cells. The mitotic spindle was stained with anti-Tubulin antibody (green) and nucleus DNA was visualized by DAPI (blue). Arrows indicated the misaligned and lagging DNA. Scale bar, 10 µm. (**c**) Silkworm cells stably expressing the histone protein EGFP-H4 were treated with indicated dsRNAs and analyzed by live-cell microscopy. RNAi of *BmSMT3*/*BmUBC9* or *BmPLK1* resulted in the failure of chromosome congression and segregation, even after a prolonged duration of mitotic delay. Scale bar, 10 µm. The percentages of silkworm cells harboring the abnormal progression were calculated in selected mitotic cells.

How Plk1 is recruited to different regions is a crucial issue for understanding the regulatory mechanism of Plk1 functions. It has been recently reported that the localization of human Plk1 in mitosis is dependent on its ubiquitylation mediated by Cullin–RING E3 ubiquitin ligase CUL3–KLHL22 complex^14^. We thus wondered if ubiquitylation is involved in the localization of BmPlk1. We identified homologies of CUL3–KLHL22 in silkworm, which are BmCUL3 and BmKLHL. RNAi of *BmCUL3* or *BmKLHL* resulted in the misalignment of chromosome at metaphase (Fig. 2a), and a similar defect was also observed in *BmCUL4B*-depleted cells, another Cullin–RING E3 ubiquitin ligase gene (Fig. 2a). These showed together that the BmCUL3–BmKLHL and/or BmCUL4B would regulate the chromosome behaviors at metaphase mediated by BmPlk1 ubiquitylation, which was the same as human Plk1.

It was previously reported that the SUMOylation machinery is able to regulate the correct alignment of chromosome during mitosis^23^. Knockdown of the components of SUMOylation machinery, such as *BmSMT3*, *BmUBC9*, and deSUMOylation enzyme of *BmULP1* led to severe misalignment of chromosomes at metaphase (Fig. 2b), similar to *BmPLK1* RNAi. To examine the effects of these genes RNAi on chromosome behaviors in detail, we constructed a silkworm cell line stably expressing the histone marker protein of EGFP-H4. Live-cell microscopy of EGFP-H4 revealed that *BmSMT3*/*BmUBC9* or *BmPLK1* RNAi cells showed the failure of chromosome congression and segregation, even after a prolonged duration of mitotic delay, when compared to the control RNAi (Fig. 2c). Taken together, these observations have raised the possibility that the SUMOylation may affect chromosome behaviors via BmPlk1 function to a certain extent, beyond the ubiquitylation.

### SUMOylation can regulate the localization of BmPlk1 during mitosis

**Figure 3:**
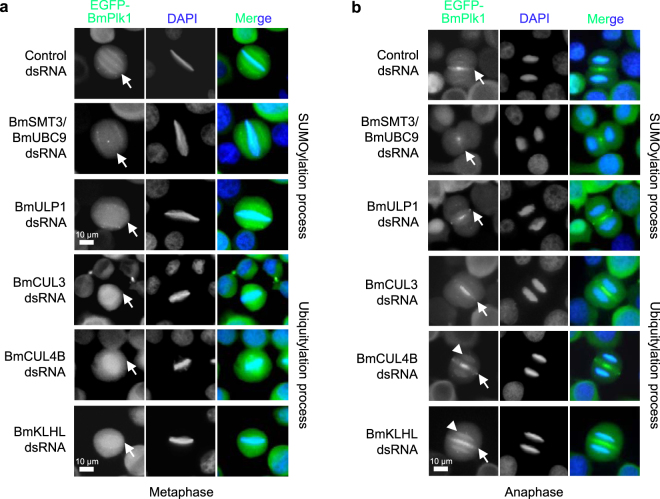
SUMOylation can regulate the localization of BmPlk1 during mitosis. (**a**) In the EGFP-BmPlk1 expressing cells, RNAi of SUMOylation genes affected the kinetochore localization of EGFP-BmPlk1 at metaphase, whereas ubiquitylation genes RNAi induced the increase of EGFP-BmPlk1 on mitotic spindle. The nucleus DNA was visualized by DAPI (blue). Arrows indicated the metaphase plate. Scale bar, 10 µm. (**b**) In the EGFP-BmPlk1 expressing cells, RNAi of SUMOylation genes and ubiquitylation genes led to the ectopic accumulation of BmPlk1 on the midzone and chromosomes. The nucleus DNA was visualized by DAPI (blue). Arrows indicated the midzone and arrowheads showed the separated chromosomes. Scale bar, 10 µm.

To understand whether the SUMOylation is involved in the regulation of BmPlk1, we investigated the localization of EGFP-BmPlk1 in the deficient cells of the SUMOylation system. At metaphase, knockdown of *BmSMT3*/*BmUBC9* or *BmULP1* affected chromosome condensation and thus resulted in the reduced and missed localization of BmPlk1 on the kinetochores (Fig. 3a), indicating that the SUMOylation process contributes to BmPlk1 localization on the kinetochores. We also observed that RNAi of ubiquitylation-related genes could affect kinetochore localization of BmPlk1 at this phase and exhibit strong fluorescent signals of BmPlk1 around the mitotic spindle (Fig. 3a). At anaphase, BmPlk1 was visualized in the spindle midzone, however, the localizations of BmPlk1 were largely decreased in the absence of BmSmt3/BmUbc9 (Fig. 3b). Interestingly, knockdown of *BmULP1* led to an accumulation of BmPlk1 at the both sides of the midzone (Fig. 3b). These results implied that the SUMOylation and deSUMOylation could cooperatively regulate the localization of BmPlk1 at the midzone. In addition, RNAi of *BmCUL3*, *BmCUL4B*, or *BmKLHL* all induced the increases of BmPlk1 at the midzone, and importantly, *BmCUL4B* or *BmKLHL*-depleted cells showed ectopic accumulation of BmPlk1 on the separated chromosomes (Fig. 3b), indicating the lack of BmPlk1 degradation by ubiquitylation-dependent pathway leads to the increases of BmPlk1 on the chromosomes, which was consistent with the previous report from human Plk1^14^. Altogether, the present data has shown that the SUMOylation system is able to regulate BmPlk1 localization during mitosis, and this regulation would be different from BmPlk1 ubiquitylation.

### SUMOylation protein BmSmt3 colocalizes with BmPlk1 at a set of structures during mitosis

**Figure 4:**
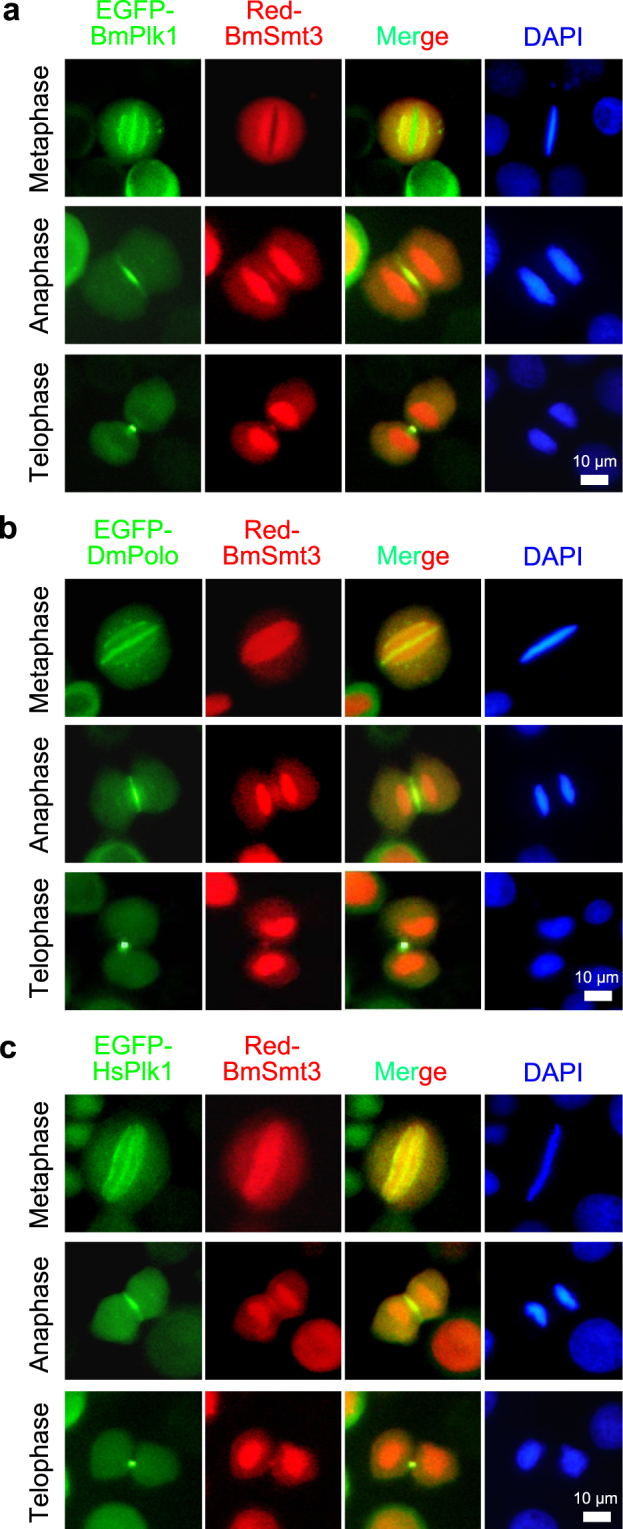
SUMOylation protein BmSmt3 colocalizes with BmPlk1 at a set of structures during mitosis. (**a**) Plasmids encoding Red-BmSmt3 and EGFP-BmPlk1 were co-transfected into the silkworm cells. After three days of transfection, cells were fixed and stained with DAPI (blue). The colocalization of BmSmt3 and BmPlk1 was shown during mitosis. Scale bar, 10 µm. Similar to the BmPlk1, the conserved localization of DmPolo (**b**) and HsPlk1 (**c**) with BmSmt3 were also observed.

To determine the temporal and spatial relationship between SUMOylation and BmPlk1, the silkworm cells were cotransfected with EGFP-BmPlk1 and Red-BmSmt3. As shown in Fig. 4a, both BmPlk1 and BmSmt3 were localized to mitotic spindle at metaphase and midzone at anaphase, as well as midbody at telophase by fluorescence microscope observation. Localization of BmSmt3 in vicinity of chromosomes at the metaphase plate may also be involved in the regulation for BmPlk1 localization on kinetochores via the interaction.

To examine whether the interaction between BmPlk1 and BmSmt3 is conserved among different species, we then analyzed the localization of BmSmt3 with DmPolo and HsPlk1. It was interesting that the localizations of DmPolo and HsPlk1 in silkworm cells possess the same profiles as BmPlk1 during mitosis (Fig. 4a–c), which demonstrated a much conserved localizations of Plk1 in different species. Consistent with the interaction between BmPlk1 and BmSmt3, we observed that DmPolo and HsPlk1 colocalized with BmSmt3 as well (Fig. 4b,c). These results further revealed a close relationship between the SUMOylation and Plk1 across species.

### BmPlk1 is SUMOylated in silkworm

**Figure 5:**
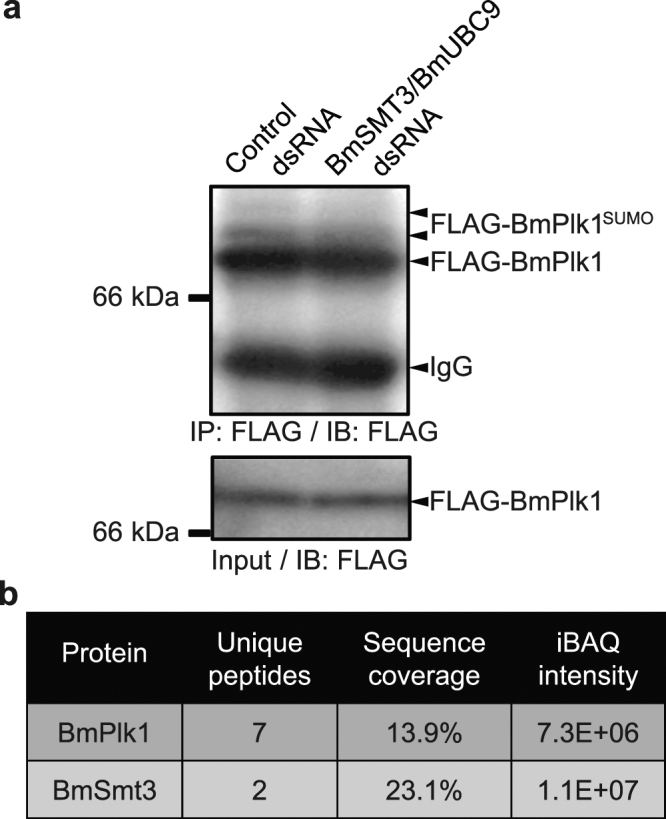
BmPlk1 is SUMOylated in silkworm. (**a**) Total lysates from FLAG-BmPlk1 cells in the presence of control dsRNA or *BmSMT3/BmUBC9* dsRNA treatments were immunoprecipitated by anti-FLAG antibody and then immunoblotted with the indicated antibody. The smeared band represented the SUMOylated FLAG-BmPlk1. Cropped blots are displayed and full-length blots can be found in Supplementary Fig. S6a,b. (**b**) Unique peptides for BmPlk1 and BmSmt3 were both identified in the smeared band by LC-MS/MS. The detailed information can be found in Supplementary Fig. S3.

The above data showed that the SUMOylation system can regulate BmPlk1 localization during mitosis. Because the ubiquitylated human HsPlk1 has been reported to involve in its localization^14^ and the amino acid residue in substrate for ubiquitylation is the same as that for SUMOylation, it was thus speculated that the regulation for BmPlk1 localization may be also mediated by the SUMOylation of BmPlk1 itself. To determine this possibility, we established a cell line that expresses FLAG-BmPlk1 protein. In the whole cell lysates, there only presented one band for FLAG-BmPlk1 detected by western blotting (Fig. 5a), which means that a small amount of protein is difficult to examine the SUMOylation. We then collected a large number of cells and performed an immunoprecipitating experiment by using FLAG-BmPlk1 as a bait to enrich the protein complex. As a result, we observed the smeared band above the FLAG-BmPlk1 in the eluates after immunoprecipitation (Fig. 5a). Moreover, when the SUMOylation genes *BmSMT3* and *BmUBC9* were knocked down, the levels of smeared band were obviously decreased (Fig. 5a). To further confirm the SUMOylation of BmPlk1 in the smeared band, we carried out LC-MS/MS analysis after specifically retrieving the band by SDS-PAGE. As shown in Fig. 5b and Supplementary Fig. S4, both of the specific unique peptides for BmPlk1 and BmSmt3 were detected in the smeared band. These data thus indicated that the BmPlk1 is SUMOylated in silkworm.

### SUMOylation of BmPlk1 regulates its localization on centrosome in interphase

**Figure 6:**
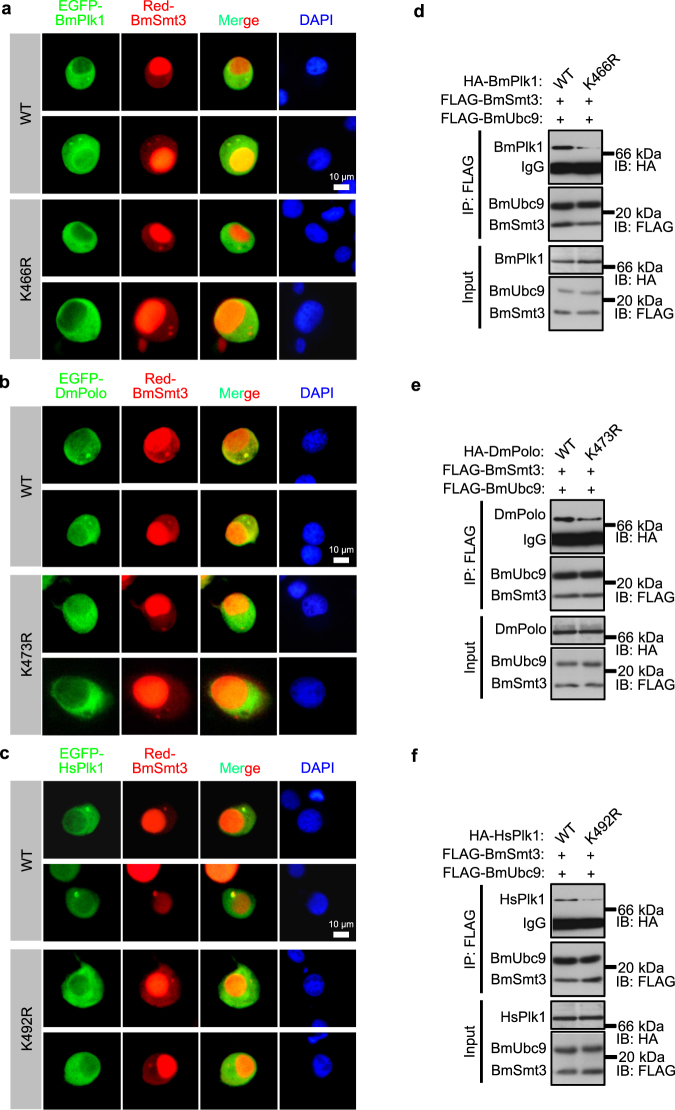
SUMOylation of BmPlk1 regulates its localization on centrosome in interphase. (**a**,**b**,**c**) The colocalization of Red-BmSmt3 with EGFP-BmPlk1, EGFP-DmPolo, and EGFP-HsPlk1 were shown on the mother centrosome and duplicated centrosomes. Mutants of EGFP-BmPlk1_K466R, DmPolo_K473R, and HsPlk1_K492R abolished this localization on the centrosomes. The nucleus DNA was visualized by DAPI (blue). Scale bar: 10 μm. (**d**,**e**,**f**) Silkworm cells transfected with the indicated vectors were subjected to coimmunoprecipitation with anti-FLAG antibody-coupled beads. Immunoblotting analyses were performed with the antibodies as shown, and revealed the strong interaction between BmSmt3/BmUbc9 and wild-type (WT) of BmPlk1, DmPolo, and HsPlk1, but weak signals in their mutations, respectively. Cropped blots are displayed and full-length blots can be found in Supplementary Fig. S7a–l.

To further explore the exact residue of BmPlk1 as the SUMOylation acceptor site, we performed an *in silico* analysis of BmPlk1 amino acid sequence, and as a result, four potential consensus SUMOylation motifs were found (Supplementary Fig. S3a). Comparison of these four motifs of BmPlk1 with DmPolo and HsPlk1 showed its conservation in a variety of species (Supplementary Fig. S3b). Importantly, lysine residue 492 of HsPlk1 has been defined as an ubiquitylation site in human and reported to involve in correct localization of HsPlk1 during mitosis^14^. We then generated mutations of the lysine residue to arginine (Supplementary Fig. S3b). As shown in Fig. 6a and Supplementary Fig. S3c, only mutation of Lys 466 was able to abrogate the localization of BmPlk1 on the centrosomes, indicating that Lys 466 of BmPlk1 may be the main target for SUMOylation. Unexpectedly, we could not detect the SUMOylated form of BmPlk1 (smeared bands similar to Fig. 5) in the immunoprecipitation assay in wild-type, K150/163R, and K328R mutations, but only the decreased interaction of BmSmt3/BmUbc9 with BmPlk1 K466R mutation (Supplementary Fig. S3d). The undetected SUMOylation of BmPlk1 here may be ascribed to insufficient amount of proteins resulted from the transient transfection for immunoprecipitation. These data, however, implied a critical role of Lys 466 for BmPlk1 interaction with BmSmt3/BmUbc9 complex. Interestingly, BmPlk1 K466R mutant cannot be recruited to duplicated centrosomes either (Fig. 6a), indicating the importance of Lys 466 for BmPlk1 centrosomal localization. Consistent with this observation, DmPolo K473R and HsPlk1 K492R mutants were not localized to centrosomes any more (Fig. 6b,c). Moreover, Lys 466 in BmPlk1, Lys 473 in DmPolo, and Lys 492 in HsPlk1 were all involved in the interaction with the SUMOylation components of BmSmt3 and BmUbc9 (Fig. 6d–f). Taken together, these data showed a physical interaction of Plk1 with SUMOylation components during the cell cycle, and that the SUMOylation of BmPlk1 at Lys 466 would regulate its centrosomal localization.

### SUMOylation of BmPlk1 at Lys 466 regulates its accurate localization during cell cycle

**Figure 7:**
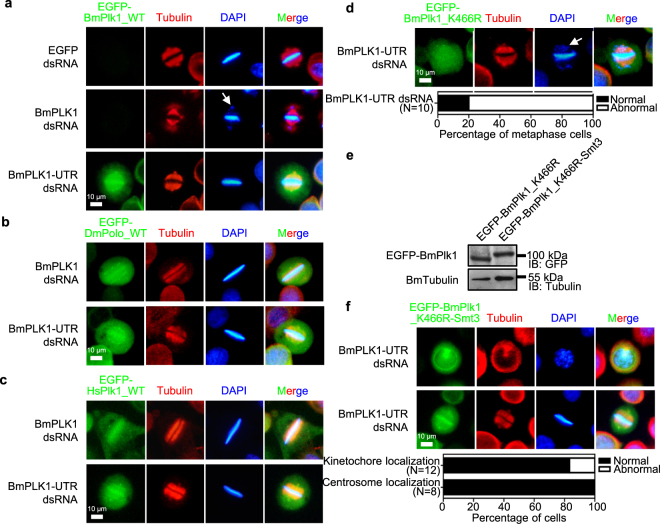
SUMOylation of BmPlk1 at Lys 466 regulates its accurate localization during cell cycle. (**a**) Silkworm cells expressing EGFP-BmPlk1_WT was able to recover the proper alignment of chromosomes on the metaphase plate in the absence of endogenous BmPlk1. The mitotic spindle was stained with anti-Tubulin antibody (red) and nucleus DNA was visualized by DAPI (blue). Arrows indicated the misaligned and lagging DNA. Scale bar, 10 µm. Both of EGFP-DmPolo_WT (**b**) and EGFP-HsPlk1_WT (**c**) could also rescue the defects of chromosome alignment caused by *BmPLK1* depletion. (**d**) Mutant of EGFP-BmPlk1_K466R lost the kinetochore localization and chromosome alignment in the absence of endogenous BmPlk1. The mitotic spindle was stained with anti-Tubulin antibody (red) and nucleus DNA was visualized by DAPI (blue). Arrows indicated the misaligned and lagging DNA. Scale bar, 10 µm. The percentages of silkworm cells harboring the abnormal alignments were calculated in selected phase of metaphase cells. (**e**) Immunoblotting analyses were carried out to detect the expression of EGFP-BmPlk1_K466R and EGFP-BmPlk1_K466R-Smt3, the increased size in EGFP-BmPlk1_K466R-Smt3 represents the conjugation of BmSmt3 to EGFP-BmPlk1_K466R. The level of BmTubulin was used as a loading control. Cropped blots are displayed and full-length blots can be found in Supplementary Fig. S8a,b. (**f**) In the EGFP-BmPlk1_K466R-Smt3 expressing cells, RNAi of the endogenous *BmPLK1* did not influence the centrosome and kinetochore localization of BmPlk1_K466R-Smt3 and showed the normal chromosome alignment. The mitotic spindle was stained with anti-Tubulin antibody (red) and nucleus DNA was visualized by DAPI (blue). Scale bar, 10 µm. The percentages of silkworm cells harboring the normal localizations were calculated in selected cells.

To further confirm the SUMOylation of Lys 466 that is involved in the correct localization and function of BmPlk1 during the cell cycle, we generated a series of constructs of BmPlk1 to analyze their effects on the cell cycle in the absence of endogenous BmPlk1 by using dsRNA against UTR of *BmPLK1* mRNA. It was shown that the cells expressing EGFP-BmPlk1_WT were able to recover the accurate alignment of chromosomes on the metaphase plate by knockdown of *BmPLK1-UTR* rather than by *BmPLK1* RNAi (Fig. 7a). Moreover, both of EGFP-DmPolo_WT and EGFP-HsPlk1_WT could also rescue the defects of chromosome alignment caused by *BmPLK1* depletion (Fig. 7b,c). These data thus indicated that the ectopic expression of Plk1 showed the functional kinase activity capable of correct chromosome behavior.

Because BmPlk1 K466R mutant made it missing the localization on centrosome, we next examined if it could affect the recruitment to the kinetochores in the absence of endogenous BmPlk1. As shown in Fig. 7d, the cells expressing EGFP-BmPlk1_K466R mutant could not be localized to kinetochores but with disordered distribution, thus leading to incorrect alignment of chromosomes. These results suggested that the SUMOylation of BmPlk1 at Lys 466 would play critical roles in BmPlk1 activity on kinetochore function. To clarify this, a fusion of EGFP-BmPlk1_K466R with BmSmt3 was constructed and stably expressed in silkworm cells. Expression of EGFP-BmPlk1_K466R-Smt3 was measured by immunoblotting, and the size of which was larger than EGFP-BmPlk1_K466R (Fig. 7e). Using this cell line, we found that, in the absence of endogenous BmPlk1, EGFP-BmPlk1_K466R-Smt3 was able to localize on the centrosomes in interphase and kinetochores at metaphase, which means that the conjunction of BmSmt3 with BmPlk1_K466R mutation could help the rescue of *BmPLK1* RNAi phenotype (Fig. 7f). All these data demonstrated that the SUMOylation of Lys 466 would modulate the localization of BmPlk1 during the cell cycle and thus regulate the centrosome and kinetochore activity of BmPlk1.

## Discussion

Plk1 is crucial for cell cycle progression throughout the entire cell cycle. Here, we identify this conserved mitotic kinase in the lepidopteran insect of the silkworm *Bombyx mori* and clarify its role in chromosome congression and segregation in mitosis. Importantly, we explore a potential regulatory mechanism that the SUMOylation is able to modify BmPlk1 and participates in the correct recruitment of BmPlk1 to centrosome and kinetochore.

Spatio-temporal localization of Plk1 is required for its function. Previous studies have established that CUL3-KLHL22 can catalyze human Plk1 ubiquitylation at Lys 492 so as to remove its localization at kinetochores on chromosome bi-orientation^14^. Our results also supported this conclusion that the ubiquitylation involved by BmCUL3-BmKLHL and/or BmCUL4B contributes to the dissociation of BmPlk1 from kinetochores in the silkworm cells. However, little is known about how Plk1 is recruited to kinetochores. Like ubiquitylation, the SUMOylation is able to regulate various biological pathways including protein localization^20^. Moreover, the defects of the SUMOylation system in silkworm resulted in the failure of chromosome alignment and segregation^23^. These prompted us to investigate whether the SUMOylation could regulate the localization of BmPlk1. It was interesting that our data showed the decreased localization of BmPlk1 on kinetochores at metaphase by depletion of the SUMOylation-related genes, suggesting that the SUMOylation would have roles in BmPlk1 kinetochore localization, which is different from the role of ubiquitylation system as shown in the present work and human^14^. We further demonstrated that BmPlk1 was SUMOylated in silkworm, and the reduced interaction between BmPlk1 and BmSmt3/BmUbc9 and the abolished localization of BmPlk1 on kinetochores by mutated Lys 466 revealed an important role of BmPlk1 SUMOylation for proper progression of cell cycle. Using the silkworm as a model, we also confirmed that this mechanism is conserved in *Drosophila* and human through interaction and localization assays by using their respective orthologs. Importantly, the fusion of BmSmt3 to BmPlk1_K466R mimics the normal localization on kinetochores and rescues the mitotic defects observed in the absence of endogenous BmPlk1. Taken together, our findings demonstrate that BmSmt3/BmUbc9-mediated SUMOylation promotes the localization of BmPlk1 at kinetochores on chromosome bi-orientation, whereas the ubiquitylation of BmPlk1 would lead to the dissociation from kinetochores.

In addition, we observed that the mutation of BmPlk1 at Lys 466 influences the centrosomal localization. The centrosome is the major microtubule-organizing center of most somatic cells, which are duplicated and segregated along with the genome during cell cycle^24–26^. It has been reported that the centrosomal localization of Plk1 is required for centrosome duplication, maturation, and separation^27–29^. The recruitment mechanism of Plk1 to centrosomes, however, remains largely unknown. The recent reports show that the centrosomal proteins of hCenexin1 (human cenexin 1) and FOR20 (FOP-related protein of 20 kDa) contribute to the targeting of Plk1 to centrosomes via their specific interactions^30,31^. In contrast to the previous recruitment mechanism of Plk1, our present evidence indicates that BmSmt3/BmUbc9-mediated SUMOylation of BmPlk1 is involved in targeting itself to centrosomes, which represents an uncharacterized model. In order to confirm the exact site for BmPlk1 SUMOylation, however, the further mass spectrometry analysis or *in vitro* SUMOylation reactions is worthy to do.

In conclusion, our present data reveals a SUMOylation-mediated signaling cascade that regulates the accurate localization of Plk1, which ensures the faithful transmission of genetic information. It thus provides a novel layer for the regulation of Plk1 localization and activity throughout the cell cycle, beyond the ubiquitylation. It also offers a framework for further dissection of the crosstalk between SUMOylation and ubiquitylation in cooperatively and dynamically regulating the localization of the same target protein.

## Methods

### Plasmids

Full-length cDNAs of Plk1 were amplified by PCR from cDNA libraries of *Bombyx* BmN4, *Drosophila* S2, and human HepG2 cells, and cloned into a pENTR11 vector (Invitrogen). Gateway recombination of the pENTR11 clones for constructing EGFP-BmPlk1, EGFP-DmPolo, EGFP-HsPlk1, HA-BmPlk1, HA-DmPolo, HA-HsPlk1, and FLAG-BmPlk1 were described previously^32^. EGFP-H4, Red-BmSmt3, FLAG-BmSmt3, HA-BmSmt3, FLAG-BmUbc9, and HA-BmUbc9 were from our previous report^23,33^.

To generate the Plk1 mutants, such as BmPlk1_K150/163R, BmPlk1_K328R, BmPlk1_K466R, DmPolo_K473R, and HsPlk1_K492R, PCR-based mutagenesis was carried out (Supplementary Table S1). To construct the fusion expression vector of BmPlk1_K466R and Smt3, the silkworm Smt3 gene was firstly cloned into the pENTR11 vector, and the resulted plasmid was further ligated with the PCR product of BmPlk1_K466R. All the mutant clones were sequenced to confirm the introduced mutations. Plasmids of genes for dsRNA synthesis *in vitro* were cloned into a pLits vector with T7 RNA polymerase promoters as described previously^32,34^.

### Cell lines

The silkworm BmN4-SID1 cell line that expresses a *Caenorhabditis elegans* SID-1 (*Ce*SID-1) transmembrane protein with the capacity of soaking RNAi (RNA interference) was cultured at 27 °C in IPL-41 medium (Sigma) containing 10% fetal bovine serum (FBS) (Gibco)^35^.

The expression vectors for FLAG-BmPlk1, EGFP-BmPlk1, EGFP-DmPolo, EGFP-HsPlk1, EGFP-BmPlk1_K466R, EGFP-BmPlk1_K466R-Smt3, EGFP-H4, and Red-BmSmt3 were inserted into the genome of BmN4-SID1 cell by using *piggyBac* transposition system according to the previous report^33^, and the stably transformed cells were selected by puromycin.

### RNA interference

Plasmids of pLits vectors containing BmPLK1 or other genes were used to synthesize double-stranded RNA (dsRNA) by T7 RNA polymerase *in vitro* and the RNAi treatments were performed in silkworm BmN4-SID1 cell line according to our previous strategy^32,34^.

### PCR

Total cellular RNA from RNAi treated cells was isolated and reversed using the ReveTra Ace cDNA synthesis kit in accordance with the manufacturer’s protocol (TOYOBO). The transcriptional profiles of *BmPLK1* and *BmGAPDH* genes were analyzed by semi-quantitative polymerase chain reaction (semi-quantitative PCR) using gene-specific primers (Supplementary Table S1).

### Immunofluorescence

For the analysis of fluorescently tagged proteins, cells were cultured on glass coverslips, washed once with PBS, and fixed with 4% paraformaldehyde in PBS for 10 min. For the observation of the spindle microtubules, cells were permeabilized with 0.2% Triton X-100 for 5 min, blocked for 30 min using blocking buffer (1% BSA), and stained with an anti-Tubulin (ab7291, Abcam) monoclonal antibody. The nuclei DNA were counterstained by 4′,6-diamidino-2-phenylindole (DAPI) (Invitrogen). Fluorescent images were captured by using either a Biozero microscope (BZ-8000, Keyence) or a confocal microscopy (FV1000, Olympus).

For chromosome misalignment and missegregation analysis, live cells expressing EGFP-H4 were grown on the glass bottom dishes and chromosome movements were recorded according to the green signals with an interval of 5 min by using a Biozero microscope (BZ-8000, Keyence) system. All the images were processed in ImageJ.

### Immunoprecipitation and immunoblotting

Cells were extracted in lysis buffer (50 mM Tris-HCl [pH7.5], 300 mM NaCl, 2 mM MgCl_2_, 2 mM EDTA, 1% Triton X-100, 1% sodium deoxycholate and protease inhibitors), sonicated and clarified by centrifugation at 14,000 rpm at 4 °C for 10 min. The supernatants were incubated overnight at 4 °C with anti-FLAG (F3165, Sigma) or anti-HA (sc-7392, Santa cruz biotechnology) antibody immobilized on Protein G Sepharose 4 Fast Flow (GE Healthcare). Immunocomplexes were extensively washed and analyzed by immunoblotting. Samples for western blotting were lysed in lysis buffer and analyzed by immunoblotting with anti-GFP (ab290, Abcam) or anti-Tubulin (ab7291, Abcam) antibody. The procedure for immunoblotting was performed as described previously^23,32^.

### Identification of the SUMOlyated BmPlk1 by mass spectrometry

Cells stably expressing FLAG-BmPlk1 were collected and used to perform an immunoprecipitation experiment with anti-FLAG antibody. The immunoprecipitated BmPlk1 protein complexes were eluted and separated using SDS-PAGE. The smeared band above the FLAG-BmPlk1 was retrieved and subjected to LC-MS/MS analysis by Shanghai Applied Protein Technology (Shanghai, China).

## Electronic supplementary material

Supplementary information
