# Supplementary material for: SUMOylation regulates the localization and activity of Polo-like kinase 1 during cell cycle in the silkworm, Bombyx mori
Source: Sci Rep. 2017 Nov 14;7:15536. doi: 10.1038/s41598-017-15884-7 (PMC5686133; doi:10.1038/s41598-017-15884-7)
Supplement: Supplementary file 1 — Supplementary information [file 41598_2017_15884_MOESM1_ESM.pdf]

SUMOylation regulates the localization and activity of Polo-like kinase 1 during cell cycle in the silkworm, *Bombyx mori*

Zhiqing Li[1,2], Qixin Cui[1], Jian Xu[3], Daojun Cheng[1,2], Xiaoyan Wang[1], Bingqian Li[1], Jae Man Lee[3], Qingyou Xia[1,2], Takahiro Kusakabe[3*], Ping Zhao[1,2*]

[1]State Key Laboratory of Silkworm Genome Biology, Southwest University, Chongqing, China

[2]Chongqing Engineering and Technology Research Center for Novel Silk Materials, Chongqing, China

[3]Laboratory of Insect Genome Science, Kyushu University Graduate School of Bioresource and Bioenvironmental Sciences, Fukuoka, Japan

[*]Corresponding author:

Takahiro Kusakabe: +81-92-642-2842; kusakabe@agr.kyushu-u.ac.jp

Ping Zhao: +86-23-6825-0885; zhaop@swu.edu.cn

Supplementary Information

Supplementary Figure S1. Sequence alignment of Plk1 orthologs from different species.

Comparison of amino acid sequences from Plk1 orthologs in *Homo sapiens* (HsPLK1: NP-005021), *Mus musculus* (MmPLK1: NP-035251), *Drosophila melanogaster* (DmPLK1/DmPolo: NP-524179), *Caenorhabditis elegans* (CePLK1: NP-001021174), and *Bombyx mori* (BmPLK1: NP-001296511) showed a high identity and a similar structure including one catalytic domain and two polo-box domains.

Supplementary Figure S2. Effects of RNAi for *BmPLK1* on cell proliferation in cultured silkworm cells.

Depletion of *BmPLK1* clearly inhibited the growth of silkworm cells and induced cell apoptosis. TUNEL staining was used to label apoptotic cells (green) and the nucleus DNA was visualized by DAPI (blue). Scale bar: 50 μm.

Supplementary Figure S3. LC-MS/MS protein identification of BmPlk1 and BmSmt3 from the smeared band of BmPlk1 IP product.

Supplementary Figure S4. Analysis of the putative SUMOylation sites in BmPlk1 and construction of the mutations.

(a) Four potential SUMOylation sites in BmPlk1 are predicted by online serve (http://www.abgent.com/). (b) Alignment of the conserved SUMOylation sites in BmPlk1, DmPolo, and HsPlk1. The constructs for their mutation were shown above the sequence. (c)

The cytoplasm localization of Red-BmSmt3 in interphase was used to label the centrosome. It was shown the colocalization of Red-BmSmt3 with EGFP-BmPlk1_WT, EGFP-BmPlk1_K150/163R, and EGFP-BmPlk1_K328R on the centrosome marked by arrows. Mutant of EGFP-BmPlk1_K466R lost the localization on the centrosome. The nucleus DNA was visualized by DAPI (blue). Scale bar: 10 μm. (d) Silkworm cells transfected with the indicated vectors were subjected to coimmunoprecipitation with anti-HA antibody-coupled beads. Immunoblotting analyses were performed with the antibodies as shown, and revealed the strong interaction between BmSmt3/BmUbc9 and WT, K150/163R, K328R of BmPlk1, but weak signals in the K466R mutation.

Supplementary Figure S5. Full-length gels and immunoblots relating to Figure 1 are shown.

Supplementary Figure S6. Full-length immunoblots relating to Figure 5 are shown.

Supplementary Figure S7. Full-length immunoblots relating to Figure 6 are shown.

Supplementary Figure S8. Full-length immunoblots relating to Figure 7 are shown.

Supplementary Table S1. List of primers used in this study.

Supplementary Figure S1

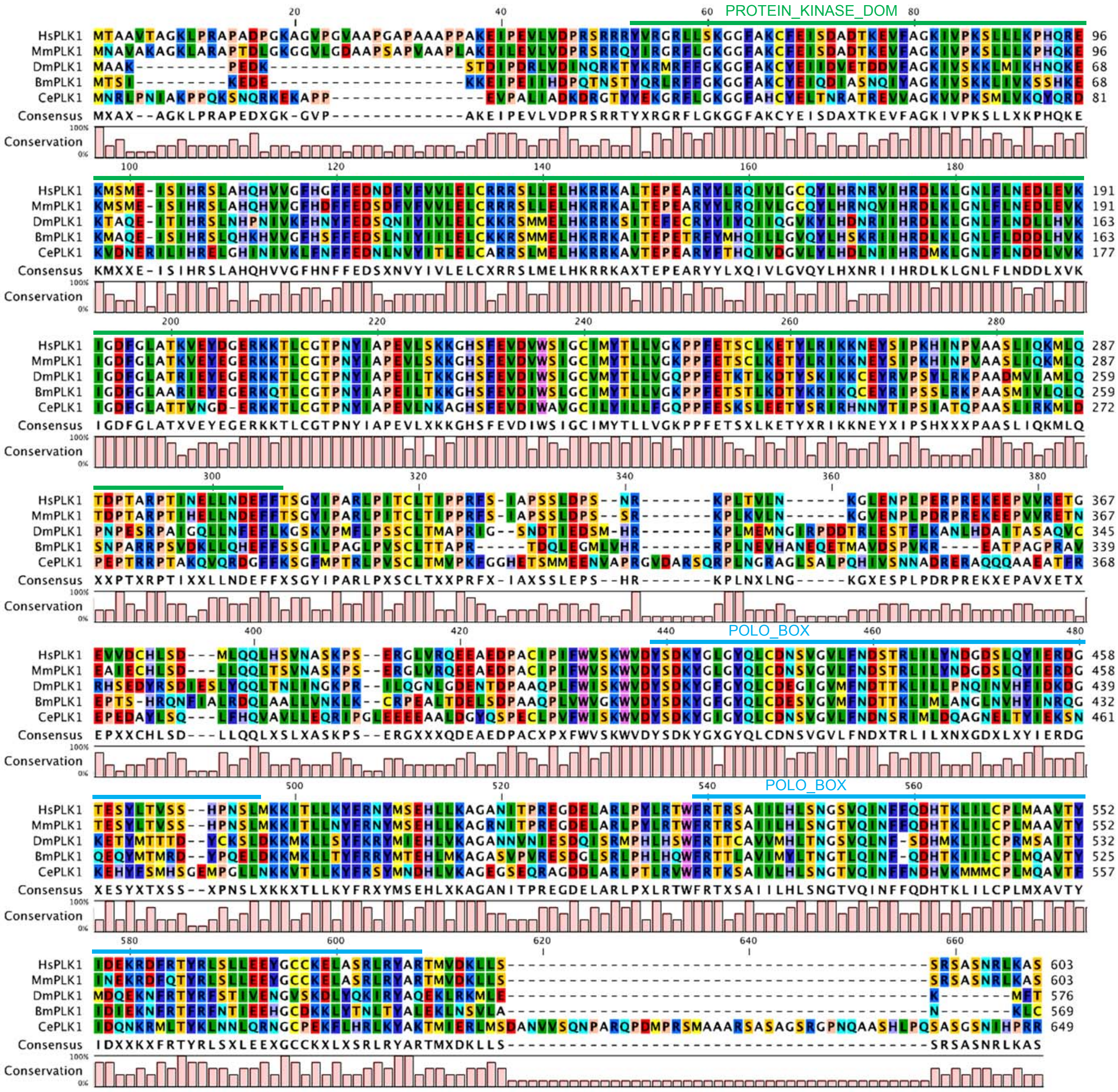

Supplementary Figure S2

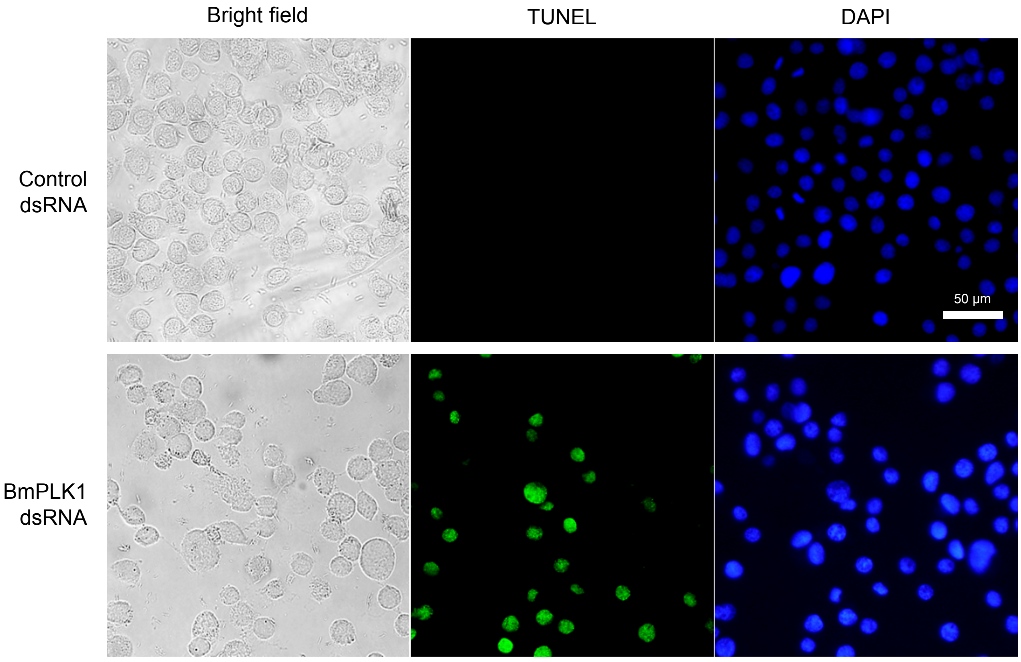

Supplementary Figure S3

BmPlk1
MTSIKEDEKKEIPEIIHDPQTNSTYQRLRFFGKGGFAKCYEIQDIASNQIYAGKIVSKKLIVKSSHKEKMAQEISIHR
SLQHKHVVGFHSFFEDSLNIYIILELCKKRSMMELHKRRKAITEPETRFYMHQILLGVQYLHSKRIIHRDLKLGNL
FLDDDLHVKIGDFGLAARIEYEGERKQTLCGTPNYIAPEILTKKGHSFEVDIWSLGCIMYTLLVGKPPFETSTLKD
TYKRIKQCEYRIPSSLRKPAASMIVLQLQSNPARRPSVDKLLQHEFFSSGILPAGLPVSCLTTAPRTDQLEGMLV
HRRPLNEVHANEQETMAVDSPVKREATPAGPRAVEPTSHRQNFIALRDQLAALLVNKLKCRPEALTDELSDPA
AQPLVWVGKWVDYSDKYGFGYQLCDESVGVMFNDTTKLIMLANGLNVHYINRQGQEQYMTMRDYPQELDKK
MKLLTYFRRYMTEHLMKAGASVPVRESDGLSRLPHLHQWFRTTLAVIMYLTNGTLQINFQDHTKIILCPLMQAV
TYIDIEKNFRTFRFNTIEEHGCDKKLYTNLTYALEKLNSVLANKLC

Unique MS spectra	Unique peptides
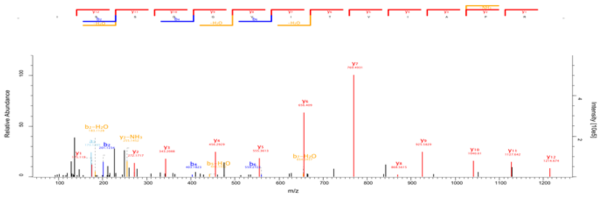	IGDFGLAAR<br>Mascot ion score: 97.43
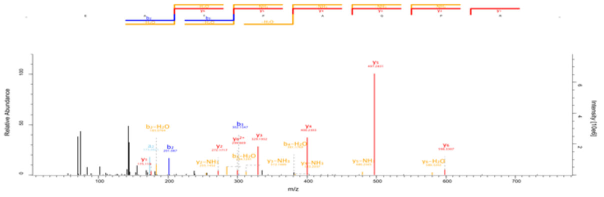	EATPAGPR<br>Mascot ion score: 113.89
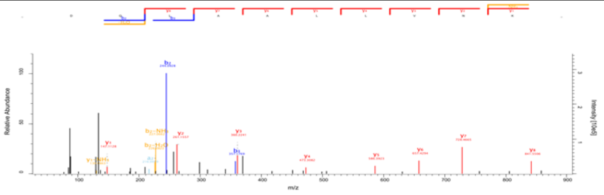	DQLAALLVNK<br>Mascot ion score: 79.97
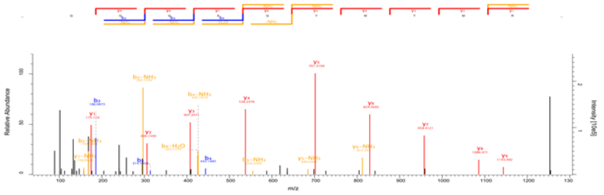	QGQEQYMTMR<br>Mascot ion score: 115.57
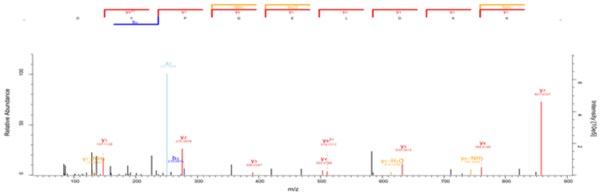	DYPQELDKK<br>Mascot ion score: 96.33
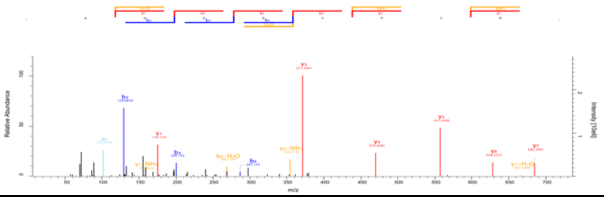	AGASVPVR<br>Mascot ion score: 109.86
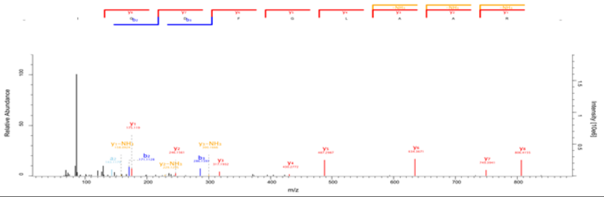	LYTNLTYALEK<br>Mascot ion score: 69.30

Supplementary Figure S3

<table>
<tr><td colspan="2">BmSmt3
MADEKKGENEHINLKVLGQDNAIVQFKIKKHTPLRKLMNAYCDRAGLSMQVVRFRFDGQPINENDTPTSLEME
EGDTIEVYQQQTGGVSLV</td></tr>
<tr><td>Unique MS spectra</td><td>Unique peptides</td></tr>
<tr><td>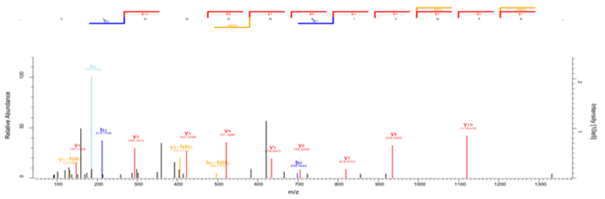</td><td>VLGQDNAIVQFK

Mascot ion score: 90.65</td></tr>
<tr><td>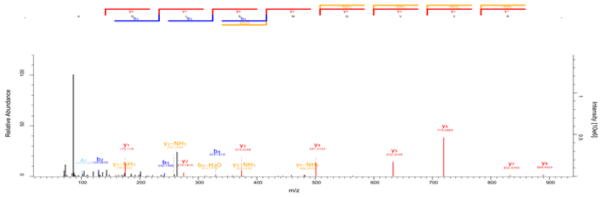</td><td>AGLSMQVVR

Mascot ion score: 106.60</td></tr>
</table>

Supplementary Figure S4

a

Residue	Score	Probability
K150	0.73	high
K163	0.76	high
K328	0.93	high
K466	0.63	high

b

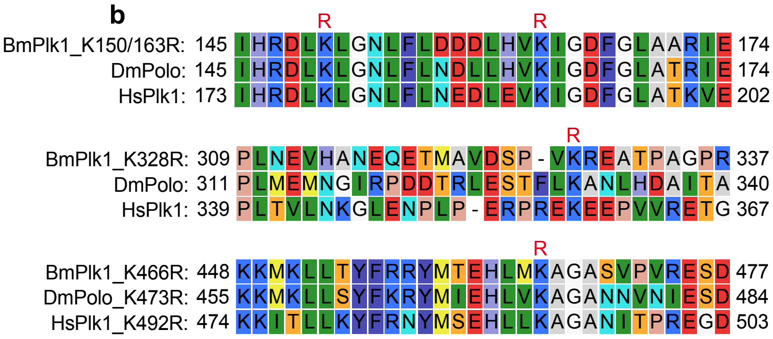

c

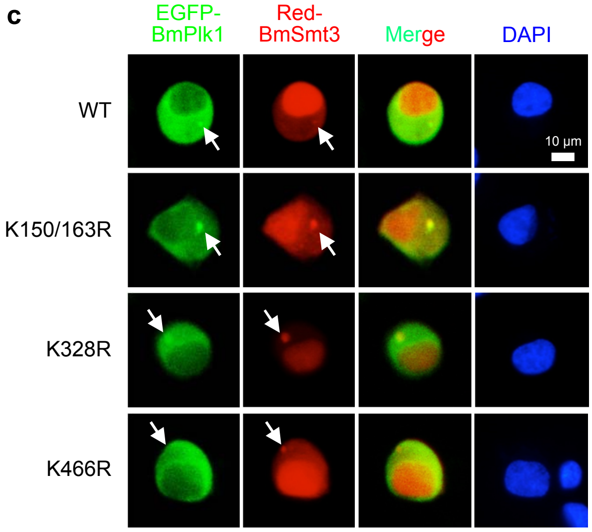

d

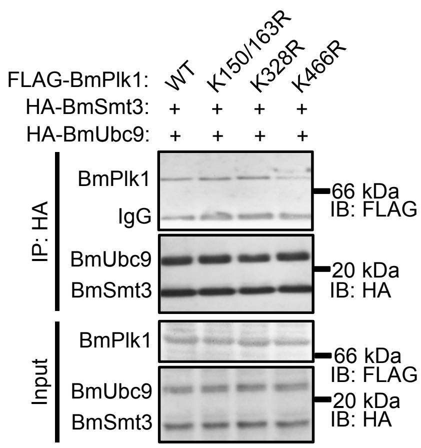

Supplementary Figure S5

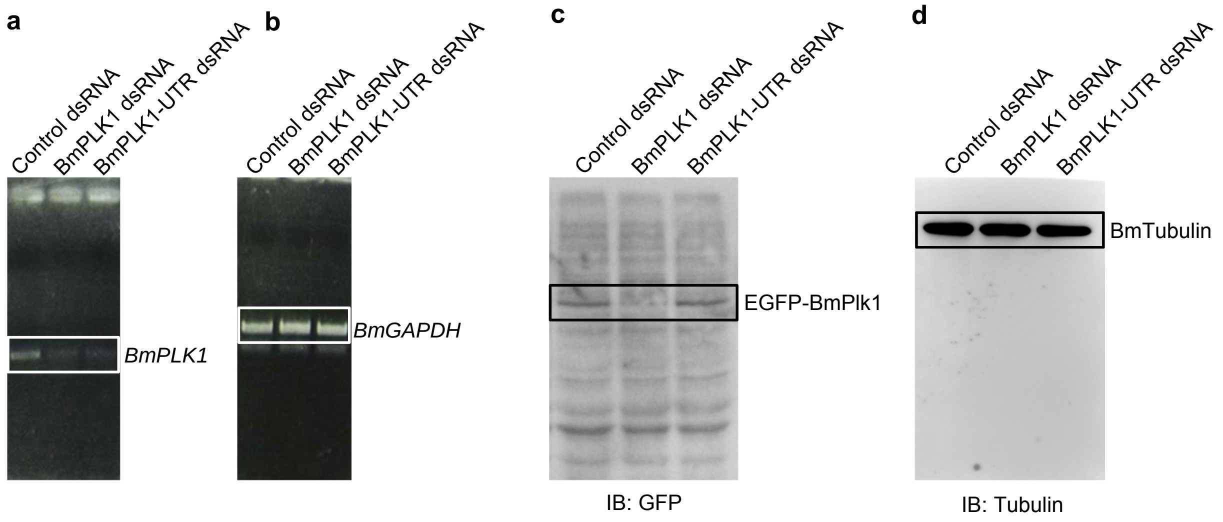

Supplementary Figure S6

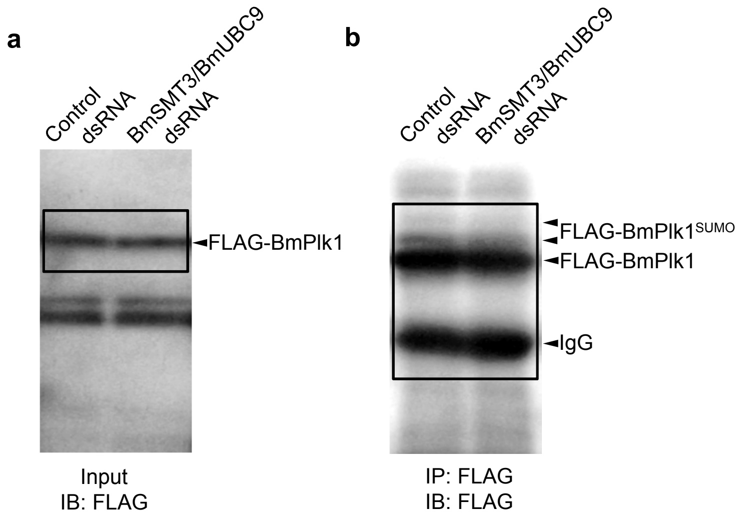

Supplementary Figure S7

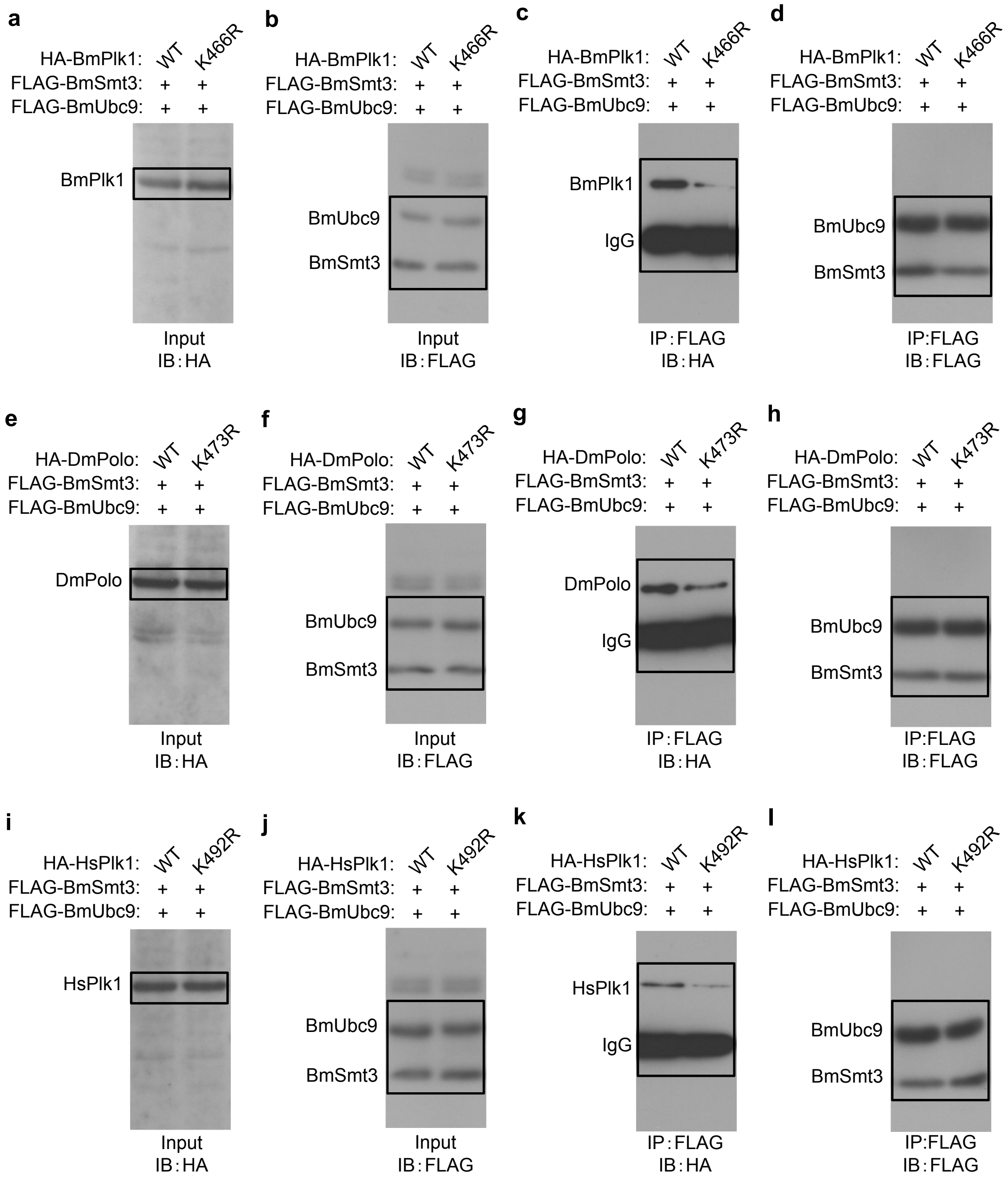

Supplementary Figure S8

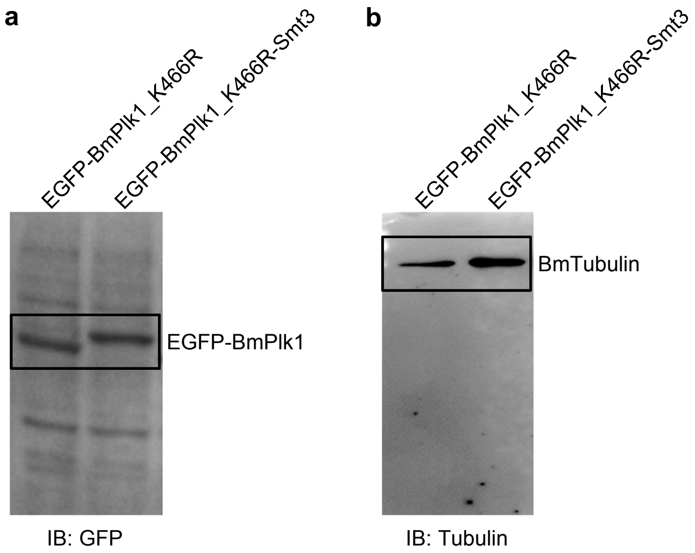

Table S1. List of primers used in this study.

Name	Sense (5' to 3')	Antisense (5' to 3')
**For cDNA clone**		
BmPLK1	ACGTCGATCAAGGAAGATGAAAAGAAAGAA	TTgcggccgccTAGCACAGTTTGTTTGCCAA
DmPolo	GCCGCGAAGCCCGAGGATAAGAGCACGGAT	CCctcgagTTATGTGAACATCTTCTCCAGCATTTTCC
HsPLK1	AGTGCTGCAGTGACTGCAGGGAAGC	CCctcgagTTAGGAGGCCTTGAGACGG
BmSMT3	GCTGATGAAAAGAAGGGAGAAAACGAACAC	CCGctcgagTTACACTAGGGACACTCCTC
BmUBC9	TCAGGGATAGCAAGTGCACGTTTAGCTG	CCGctcgagTTACTCAGCAGCAGCCATTG
BmH4	ACCGGTCGCGGTAAAGGAGGCA	CCGctcgagTTAACCGCCGAAACCGTACAGGGTGC
**For RT-PCR**		
BmPLK1	TGGATATCTGGAGTCTGGGC	CTGGTCTCGCAGTGCAATAA
BmGAPDH	GGCCGCATTGGCCGTTTGGTGCTCCG	GTGGGGCAAGACAGTTTGTGGTGCAAGAAG
**For RNAi**		
BmPLK1	TGGATATCTGGAGTCTGGGC	CTGGTCTCGCAGTGCAATAA
BmPLK1-UTR	gcgtaatacgactcactatagggCACTGAACCTAAATGAGTAAAT	gcgtaatacgactcactatagggGAAAGTTTTGCTCATATACTGA
BmSMT3	GCTGATGAAAAGAAGGGAGAAAACGAACAC	CCGctcgagTTACACTAGGGACACTCCTC
BmUBA2	CTCCACCTAATCCTAAGTGCTATGTCTG	CCGctcgagTTAACATATTTCGACAACTTCATC
BmULP1	TGCTAATGCAGCGAAGCCAGGATAACAAAG	TTTgcggccgccTCACAGCAGCAGGGTGC
BmCUL3	CTCTTACTCAATAAGTCTGTCTCTG	TCCCTTCGCGGCTACCGTTTGTATC
BmCUL4B	AGTAGGTAAATCTGCATCGGTGGACG	GAACAGCTTGTTGGTGAAGTCCGCA
BmKLHL	TCAACACCGTAGAGCGGTACGAC	AACCGCAGTAGAGTCGTCGAACACC
dsEGFP	ATTTGCACTACTGGAAAACTACCTG	CAGTTACAAACTCAAGAAGGACCAT
dsLUC	GAAGCGACCAACGCCTTGATTGACAAGGAT	TTACAATTTGGACTTTCCGC
T7	gcgtaatacgactcactataggg	
**For mutation assay**		
BmPLK1-K150/163R	GATGACGATCTTCATGTACGAATTGGAGATTTTGGTCTTGC	AAGGAACAAATTGCCAAGTCGCAAGTCTCGATGTATTATCC
BmPLK1-K328R	GCTACCCCCGCGGGGCCACGA	TTCGCGTCGGACGGGCGAGTCCACT
BmPLK1-K466R	CTCATGCGAGCTGGTGCTTCAGTACCA	ATGTTCGGTCATGTATCGTCTAAAG
DmPolo-K473R	CACCTGGTGCGAGCAGGTGCCAACAAT	CTCGATCATGTAGCGCTTAAAGTA
HsPLK1-K492R	CTGCGAGCAGGTGCCAACATCACG	CAAGTGCTCGCTCATGTAATTGCG
